# Utility of urine and serum lateral flow assays to determine the prevalence and predictors of cryptococcal antigenemia in HIV-positive outpatients beginning antiretroviral therapy in Mwanza, Tanzania

**DOI:** 10.7448/IAS.17.1.19040

**Published:** 2014-08-08

**Authors:** Kinanga A Magambo, Samuel E Kalluvya, Shikha W Kapoor, Jeremiah Seni, Awilly A Chofle, Daniel W Fitzgerald, Jennifer A Downs

**Affiliations:** 1 Department of Internal Medicine, Catholic University of Health and Allied Sciences Bugando, Mwanza, Tanzania; 2 Department of Microbiology and Immunology, Catholic University of Health and Allied Sciences Bugando, Mwanza, Tanzania; 3 Department of Internal Medicine, Muhimbili National Hospital, Dar-es-Salaam, Tanzania; 4 Center for Global Health, Department of Medicine, Weill Cornell Medical College, Cornell University, New York, NY, USA

**Keywords:** cryptococcal antigenemia, Cryptococcus, lateral flow assay, HIV, Mwanza, outpatient

## Abstract

**Background:**

Detection of subclinical cryptococcal disease using cryptococcal antigen screening among HIV-positive individuals presents a potential opportunity for prevention of both clinical disease and death if patients with detectable cryptococcal antigen are identified and treated pre-emptively. Recently developed point-of-care cryptococcal antigen tests may be useful for screening, particularly in resource-limiting settings, but few studies have assessed their utility.

**Methodology:**

The objectives of this study were to determine the prevalence and factors associated with cryptococcal antigenemia in HIV-positive patients with CD4^+^ T-cell counts ≤200 cells/µL who were initiating ART, and also to evaluate the utility of the point-of-care urine lateral flow assay (LFA) cryptococcal antigen test using two different diluents, compared to gold standard serum antigen testing, as a screening tool. Urine and serum of outpatients initiating antiretroviral therapy at two hospitals in Mwanza were tested for cryptococcal antigen, and demographic and clinical characteristics were obtained using structured questionnaires and patients’ files. Patients with asymptomatic cryptococcal antigenemia received oral fluconazole in accordance with World Health Organization recommendations.

**Results:**

Among 140 patients screened, 10 (7.1%) had asymptomatic cryptococcal antigenemia with a positive serum cryptococcal antigen. Four of these ten patients had CD4 counts between 100 and 200 cells/µL. The prevalence of cryptococcal antigen detected in urine using a standard (older) and a test (newer) diluent were 44 (31.4%) and 19 (13.6%), with Kappa coefficients compared to serum of 0.28 and 0.51 (*p*<0.001 for both). Compared to the new LFA diluent for urine cryptococcal antigen, the standard diluent had higher sensitivity (100% versus 80%) but lower specificity (74% versus 92%) using serum cryptococcal antigen as a gold standard.

**Conclusions:**

Our findings suggest that HIV-positive outpatients with CD4 counts <200 cells/µL, rather than 100, should be screened for asymptomatic cryptococcal antigenemia given its association with mortality if untreated. Agreement of the urine LFA with the serum LFA was not sufficient to recommend routine screening with urine LFA.

## Introduction

*Cryptococcus neoformans* is one of the leading opportunistic infections in people living with HIV/AIDS in sub-Saharan Africa [1–4]. It is estimated that 3% of HIV-positive individuals in sub-Saharan Africa develop cryptococcal meningitis (CM) each year with mortality rates as high as 75% in settings in which amphotericin B-based treatments are not available and many patients with CM are also newly diagnosed with HIV and have not yet started antiretroviral therapy (ART) [5–9]. Furthermore, CM can present as an immune reconstitution inflammatory syndrome (IRIS) following the initiation of ART in patients with subclinical cryptococcal disease [3, 6]. In a South African study, CM IRIS was implicated in 20% of all deaths of patients within the first four months of ART initiation, which was more common than TB-attributable mortality [6].

Even when managed optimally, the mortality rate of CM is likely ~15–25% [10, 11], and the substantially higher mortality rate in sub-Saharan Africa highlights the necessity of preventing the development of disease. Of note, the clinical course of cryptococcal disease provides a window, lasting a median of 22 days [5], during which cryptococcal antigen (CrAg) can be detected in serum but patients have not yet developed symptoms and clinical disease [7, 12, 13]. This condition, known as asymptomatic cryptococcal antigenemia, has been associated with early mortality among those with advanced HIV [14] and was an independent predictor of mortality in patients initiating ART in Uganda [12]. Identification and pre-emptive fluconazole treatment of asymptomatic CrAg-positive patients has been endorsed by the World Health Organization (WHO) and others as a cost-effective opportunity for prevention of both clinical disease and death due to cryptococcal disease [7, 13, 15, 16].

The CrAg assay has recently been developed into a point-of-care lateral flow assay (LFA), which can test serum, blood, cerebrospinal fluid, or urine, for approximately $2.50 per test. The LFA test was shown to correlate highly with traditional cryptococcal antigen testing (i.e. enzyme immunoassay) in the serum, plasma, and urine of patients with CM [16–18], and has been noted by the WHO to meet most of its ASSURED criteria for point-of-care tests [15]. The LFA test has been evaluated as a screening test in serum but not in urine in asymptomatic patients [19]. In sub-Saharan Africa, where the prevalence of HIV is high and laboratory resources are limited, the need for rapid and effective point-of-care screening for CrAg among HIV-positive outpatients who are starting ART is urgent and, if made available, has the potential to decrease mortality. Optimization of a urine test would make screening possible even in remote health posts, where blood collection may be challenging.

Therefore, in the present study, our objective was to determine the utility of the LFA CrAg test using two different diluents in urine, compared to serum, for screening of HIV-positive outpatients in Tanzania. We hypothesized that the correlation between urine and serum LFA would be excellent given the high correlations between urine and serum LFA results reported for patients with CM [18]. A second objective was to describe the prevalence and clinical factors associated with asymptomatic cryptococcal antigenemia in order to guide CrAg screening recommendations.

## Methodology

### Study design and sampling process

This cross-sectional study was conducted at the outpatient clinics of Bugando Medical Centre (BMC) and Sekou Toure regional hospital from September 2012 to March 2013. BMC is a referral, consultant and teaching hospital located in Mwanza Region, Northeastern Tanzania. The hospital serves a catchment area of ~13 million people from seven regions around the Lake Victoria Zone. Sekou Toure, located in Mwanza city, provides care to ~4 million people. Both hospitals have active care and treatment centres providing outpatient care to patients living with HIV, and each clinic initiates an average of 15 patients per week on ART.

We serially invited all HIV-positive adults (≥18 years) with CD4^+^ T-cell counts (CD4 counts) ≤200 cells/µL who were undergoing counseling sessions prior to starting ART to participate in this study. Patients already receiving fluconazole treatment, with a previous history of CM, or planning to transfer their care to another clinic were excluded from the study.

### Data collection

Socio-demographic information was collected via a structured questionnaire, and patient files were used to obtain height, weight, WHO HIV stage, CD4 count, history of previous diagnosis of CM, details of past ART exposure and history of using fluconazole. Patients were asked whether they had any symptoms of CM including fever, headache, neck stiffness, photophobia, vomiting, and altered mental status. A physical examination (including careful neurological examination and skin examination) was performed in all study participants to look for obvious signs and symptoms of cryptococcal infection. Any patients with symptoms or signs of CM underwent lumbar puncture. All patients who were serum positive for cryptococcal antigen had lumbar punctures to rule out meningitis. Patients were diagnosed with CM by positive India ink (Pelikan, Hanover, Germany) and/or CrAg (Immuno-Mycologics, Inc. (IMMY), Oklahoma, USA) in the CSF. All positive CM cases were admitted to the hospital and treated according to Tanzanian national guidelines, which recommend amphotericin B and 5-flucytosine as the “preferred” regimen but suggest an alternative of fluconazole 1200 mg IV daily when the preferred regimen is not locally available [20]. Patients admitted to Bugando Medical Centre typically receive fluconazole 1200 mg daily together with serial lumbar punctures to reduce intracranial pressure, in accordance with hospital guidelines.

## Laboratory procedures

### Lateral flow cryptococcal antigen assay

Urine and blood samples were collected from every participant. The urine samples were screened for CrAg using LFA (Immuno-Mycologics Inc., Oklahoma, USA) in all patients using both a standard and a new test urine diluent as per manufacturer's instruction. The LFA uses immunochromographic test strips that have been impregnated with monoclonal antibodies against capsular polysaccharide antigens common to fungi in the *Cryptococcal* species complex (including *C. neoformans* and *C. gattii*) [17]. To perform the assay, 40 microliters of urine or serum are mixed with one drop of the specimen diluent provided by the manufacturer. The test strip is placed into the mixture and read after ten minutes at room temperature. The presence of two lines (test and control) on the test strip regardless of the intensity of the test line were interpreted as positive result, while the presence of a single control line was read as a negative result.

The standard diluent included with the LFA kit had been previously reported to have high sensitivity [17] but low specificity; a new test diluent was engineered by the company to improve specificity by altering the buffer and adding non-ionic surfactants designed to reduce nonspecific binding.

### CD4 counts and other routine investigations

CD4 counts and other routine investigations were performed according to standard operating procedures in the hospital laboratory. Blood samples for CD4 were collected using ethylenediaminetetraacetic acid (EDTA) tubes and stored at room temperature (22–27°C). These samples were analyzed within 48 hours using an automated FACSCalibur Flow Cytometry machine (Becton Dickinson, San Jose, USA).

## Data analysis and presentation

Data were entered into Microsoft Excel and exported to Stata/IC version 11 (College Station, Texas, USA) for analysis according to the study objectives. Continuous variables were described as medians (interquartile ranges). Categorical variables were described as proportions and frequencies were compared between CrAg-positive and negative groups using Chi-squared or Fisher's exact tests where appropriate. Agreement between the urine and serum LFAs was calculated using the Cohen's kappa coefficient. Differences were considered significant if *p*-values were less than 0.05.

## Ethical issues

Study ethical clearance was obtained from the BMC/Catholic University of Health and Allied Sciences/Sekou Toure Ethics Committee and Weill Cornell Medical College. All study subjects, or their health care proxies for those with altered mental status, provided written informed consent for study participation.

All results were made available immediately to the attending clinicians and recorded in the patient's medical record. Patients with CM were managed according to the existing management protocols as described above [20]. In accordance with WHO guidelines, all patients found to have asymptomatic cryptococcal antigenemia were initiated on high-dose oral fluconazole and enrolled in an on-going operational cohort study being performed at Bugando Medical Centre. Patients screened in this study were those undergoing counseling to begin ART, which was subsequently initiated in all of these patients that same day according to the Tanzanian National Guidelines [20].

## Results

### Study population

**Figure 1:**
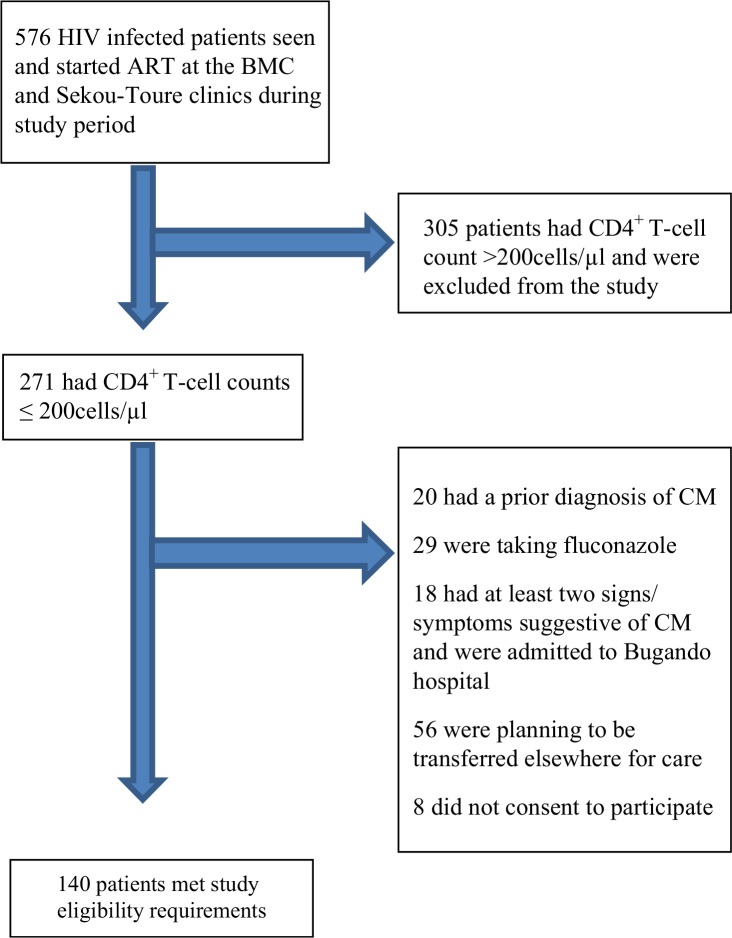
Recruitment and enrolment of patients for cryptococcal antigenemia screening.

A total of 576 patients due to start ART were seen at the BMC and Sekou Toure clinics between September 2012 and March 2013. Of these, 371 had CD4 counts >200 cells/µL, 56 were planning to be transferred elsewhere for care, and eight did not consent to participate. Eighteen patients had two or more signs/symptoms consistent with CM and were admitted directly to the hospital for diagnostic lumbar puncture and further treatment, of which six were diagnosed with confirmed CM. Thus we were left with 140 patients who met the study eligibility requirements and were enrolled (Figure 1).

The median age (IQR) of the studied population was 36 (30–42) years, and 81/140 (57.9%) were female. Median CD4 count was 97 cells/µl (IQR 49–151), and approximately half of the study population (72 patients, 51%) had CD4 counts of ≤100 cells/µl. Fifty-three patients (37.9%) were in WHO clinical stage three presentation, with 38 patients (27.1%) in stage two (Table 1).

**Table 1:** Demographic and clinical characteristics of 140 HIV-positive patients initiating antiretroviral therapy in Mwanza

Variables	Median (IQR) or number (%)
Age (years)	36 (30–42)
Sex	
Male	59 (42.1%)
Female	81 (57.9%)
Reported symptoms	
Headache	14 (10.0%)
New skin rash**	14 (10.0%)
Cough	12 (8.6%)
Reported fever	7 (5.0%)
Weight loss	6 (4.3%)
Fatigue	6 (4.2%)
Pleuritic chest pain	3 (2.1%)
Nausea	3 (2.1%)
Others*	9 (6.4%)
Presence of at least one symptom	35 (25.0)
Median CD4+ T cell count (cells/µl)	97 (49–151)
CD4 count 0–100	73 (52.0%)
CD4 count 101–200	67 (48.0%)
Body mass index (kg/m^2^)	20.5 (18.3–23.0)
Temperature above 38°C at time of enrolment	3 (2.1)
World Health Organization (clinical stage)	
I	24 (17.1%)
II	38 (27.1%)
III	53 (37.9%)
IV	25 (17.9%)

* Others: Night sweats (2), diarrhea (2), vomiting (2), increased sputum production (1), shortness of breath (1), photophobia (1).

** Skin was examined for any rash but all 10 rashes identified were consistent with pruritic papular eruption.

Headache and pruritic papular eruptions were the most common symptoms, with each reported by 14 (10.0%) patients. A total of 35/140 patients reported at least one symptom.

### Prevalence of cryptococcal antigen positivity in urine using standard (old) diluent and test (new) diluent LFA

The prevalence of asymptomatic cryptococcal antigenemia as defined as a positive serum CrAg with a lumbar puncture negative for CM was 7.1% (10/140). Six of the 73 patients with CD4 counts ≤100 cells/µL had a positive serum CrAg (8.2%), while 4/67 with CD4 counts between 100 and 200 were positive (6.0%). These four CD4 counts were 115, 153, 167, and 183 cells/µL.

Using the urine LFA, the prevalence of CrAg positivity was 44/140 (31.4%) for the standard diluent and 19/140 (13.6%) for the new diluent. Compared to serum CrAg as the gold standard, the standard urine diluent had a sensitivity of 100%, specificity of 73.8%, positive predictive value (PPV) of 22.7% and negative predictive value (NPV) of 100%, whereas the new urine diluent had a sensitivity of 80%, specificity of 91.5%, PPV of 42.1% and NPV of 98.3% (Table 2). Kappa coefficients were 0.28 for agreement between serum and the standard diluent and 0.51 for serum and the test diluent (*p*<0.001 for both).

**Table 2:** Distribution of results by standard and test urine diluents versus serum CrAg positive among 140 HIV-positive patients initiating antiretroviral therapy in Mwanza

	Serum CrAg						
						
	Positive	Negative	Total	Sensitivity (%)	Specificity (%)	Positive predictive value (%)	Negative predictive value (%)
Standard urine diluent							
Positive	10	34	44				
Negative	0	96	96	100	73.8	22.7	100
Total	10	130	140				
Test urine diluent							
Positive	8	11	19				
Negative	2	119	121	80	91.5	42.1	98.3
Total	10	130	140				

### Factors associated with asymptomatic cryptococcal antigenemia

We found no statistically significant associations between demographic or clinical factors (age, body mass index, CD4 count and WHO stage) and positive serum CrAg. The association between the headache and serum CrAg positivity trended towards significance (*p*=0.06 (Table 3)). Also of note, three of the four patients who had CD4 counts >100 cells/µL had symptoms (two with headache and one with fever and fatigue).

**Table 3:** Factors associated with positive serum CrAg among 140 HIV-positive outpatients initiating antiretroviral therapy in Mwanza

	Outcome (serum CrAg result)		
		
Risk factor	Positive, n (%) n=10	Negative, n (%) n=130	*p*
Median age (years)	42 (34–50)	36 (30–42)	0.09
Sex			
Male	4 (40.0%)	55 (42.3%)	1.0
Female	6 (60.0%)	75 (57.7%)	
Reported symptoms			
Headache	3 (30.0%)	11 (8.5%)	0.063
New skin rash	0	10 (10.8%)	0.607
Cough	1 (10.0%)	11 (8.5%)	1.0
Fever	1 (10.0%)	6 (4.6%)	0.412
Weight loss	1 (10.0%)	5 (3.8%)	0.364
Fatigue	1 (10.0%)	8 (6.2%)	0.497
Pleuritic chest pain	0	3 (2.3%)	1.0
Nausea	0	3 (2.3%)	1.0
Median CD4 count (cells/µL)	80 (50–153)	97 (48–150)	0.75
Median BMI (kg/m^2^)	20 (18.3–23.4)	20.6 (18.3–23.0)	0.98
Temperature above 38°C at time of enrolment	1 (10.0)	2 (1.5)	0.20
WHO clinical stage			
1	3 (30)	21 (16.2)	
2	3 (30)	35 (26.9)	0.58
3	2 (20)	51 (39.2)	
4	2 (20)	23 (17.7)	

## Discussion

Among HIV-positive outpatients with CD4^+^ counts ≤200 cells/µL who were initiating ART at outpatient clinics in northwest Tanzania, the prevalence of asymptomatic cryptococcal antigenemia was 7.1% overall, and was high not only in patients with CD4 counts <100 (8.3%) and but also in those with counts between 100 and 200 (6.0%). This finding calls into question the WHO's suggestion that screening for asymptomatic CrAg can be limited to patients with CD4 counts <100 cells/µL [15]. Limiting the screening to those with CD4 counts <100 in our study would have led to missed diagnoses and pre-emptive treatment in four patients (40% of the CrAg-positive patients). Given the high mortality rate associated asymptomatic (untreated) antigenemia, our findings suggest that the WHO's recommendations may need to be re-examined.

We also provide the first report, to our knowledge, of the application of the CrAg LFA test to urine for screening of asymptomatic HIV-positive outpatients. While both diluents achieved sensitivities ≥80%, both also identified numerous false positives, leading to poor PPVs (22% with standard diluent and 42% with test diluent). The LFA, when used in serum, has been recently documented to have excellent performance compared to enzyme immunoassay among inpatients [17] and to latex agglutination in another Tanzanian outpatient population [19]. In patients with CM, the urine LFA has a reported sensitivity of 98% [18]. Yet in our outpatient population, although we found a similarly high sensitivity (100% with the standard diluent), we noted an unacceptably high rate of false positives. This poor specificity would effectively negate the cost-effectiveness of the urine LFA ($18 per test when used qualitatively) [21] by necessitating approximately three confirmatory serum tests per CrAg-positive patient diagnosed.

The prevalence of cryptococcal antigenemia in this study (7.1%) is higher than rates reported from rural Uganda (5.8%) [12] and northeastern Tanzania (5%) [19] but similar to another study in South Africa (7%) [22] and also to a recently published study in Indonesia (7.1%) [14]. Other studies have shown that the diagnostic methods, host factors including prior receipt of ART, and seasonal changes may influence the prevalence in different settings [2, 12, 19, 23–25]. Studies in Uganda and Rwanda have suggested that cryptococcal environmental transmission usually peaks during the long rainy season [23, 24], which occurred after completion of our study. If cryptococcal disease in severely immunosuppressed patients is due to newly-acquired infections from the environment rather than reactivation of latent disease, then the prevalence at another time of year could in fact be higher in our setting. This further emphasizes the importance of adding routine CrAg screening to the armamentarium of recommended tests for HIV-positive patients in endemic settings. A Ugandan study estimated that the mortality in patients who have asymptomatic cryptococcal antigenemia is increased by 18% over a baseline of ~6% in the first three months after ART initiation [12]. Thus in a setting with cryptococcal antigenemia prevalence of 7%, this translates to 1 death per 67 people screened for antigenemia that can potentially be avoided through early identification and provision of pre-emptive antifungal therapy.

One possibility for expanding screening recommendations beyond patients with CD4 counts <100 cells/µL while conserving limited health care dollars could be to screen patients with higher CD4 counts if they have certain symptoms. In our study, if the screening criteria had included either those with CD4 ≤100 cells/µL or those with symptoms of fever or headache, we would have detected 9 of 10 cases of cryptococcal antigenemia. Thus, a combination approach may be a prudent cost effective strategy in a resource-limited setting to identify potential candidates for fluconazole pre-emptive therapy. Additional studies at our institution are on-going and are clearly needed to confirm our findings and to assess the effectiveness of screening and pre-emptive fluconazole treatment on mortality.

For future research on the utility of urinary LFA in determining the CrAg, we would recommend detection of the specific titer values of CrAg for those who have a positive result in their urine. In addition, we would recommend that future research ascertain whether there is an association of these urinary titer values and morbidity and mortality due to asymptomatic CM.

## Conclusions

We identified an asymptomatic cryptococcal antigenemia prevalence of 7% among HIV-positive outpatients at two hospital clinics in northwest Tanzania. Antigenemia was found in patients with CD4 counts below 100 but also those with CD4 counts between 100 and 200, suggesting that screening recommendations may need expansion to maximize detection of patients in whom fluconazole pre-emptive therapy may be life-saving. The LFA test in serum appears to be superior to LFA test in urine for screening due to inadequate specificity of the LFA in urine. Future studies of the urine LFA should investigate whether the specificity is improved in patients with highest serum CrAg titers, as well as the operational use of the urine LFA for diagnosis of CM in extremely resource-limited settings, in which obtaining CSF and even serum may be challenging.
